# Effects of therapeutic probiotics on modulation of microRNAs

**DOI:** 10.1186/s12964-020-00668-w

**Published:** 2021-01-11

**Authors:** Amirhossein Davoodvandi, Havva Marzban, Pouya Goleij, Amirhossein Sahebkar, Korosh Morshedi, Samaneh Rezaei, Maryam Mahjoubin-Tehran, Hossein Tarrahimofrad, Michael R. Hamblin, Hamed Mirzaei

**Affiliations:** 1 Student Research Committee, Kashan University of Medical Sciences, Kashan, Iran; 2 Department of Veterinary Pathology, Faculty of Veterinary Medicine, University of Tehran, Tehran, Iran; 3 Department of Genetics, Faculty of Biology,Sana Institute of Higher Education, Sari, Iran; 4 Biotechnology Research Center, Pharmaceutical Technology Institute, Mashhad University of Medical Sciences, Mashhad, Iran; 5 Neurogenic Inflammation Research Center, Mashhad University of Medical Sciences, Mashhad, Iran; 6 Faculty of Medicine, Kashan University of Medical Sciences, Kashan, Iran; 7 Student Research Committee, Mashhad University of Medical Sciences, Mashhad, Iran; 8 Department of Medical Biotechnology, Faculty of Medicine, Mashhad University of Medical Sciences, Mashhad, Iran; 9 Department of Animal Biotechnology, National Institute of Genetic Engineering and Biotechnology (NIGEB), Tehran, Iran; 10 Wellman Center for Photomedicine, Massachusetts General Hospital, Harvard Medical School, 40 Blossom Street, Boston, MA 02114 USA; 11 Research Center for Biochemistry and Nutrition in Metabolic Diseases, Institute for Basic Sciences, Kashan University of Medical Sciences, Kashan, Iran

**Keywords:** MicroRNAs, Probiotics, Biomarkers, Cancer, Inflammatory bowel disease, Supplements

## Abstract

Probiotics are beneficial bacteria that exist within the human gut, and which are also present in different food products and supplements. They have been investigated for some decades, due to their potential beneficial impact on human health. Probiotics compete with pathogenic microorganisms for adhesion sites within the gut, to antagonize them or to regulate the host immune response resulting in preventive and therapeutic effects. Therefore, dysbiosis, defined as an impairment in the gut microbiota, could play a role in various pathological conditions, such as lactose intolerance, gastrointestinal and urogenital infections, various cancers, cystic fibrosis, allergies, inflammatory bowel disease, and can also be caused by antibiotic side effects. MicroRNAs (miRNAs) are short non-coding RNAs that can regulate gene expression in a post-transcriptional manner. miRNAs are biochemical biomarkers that play an important role in almost all cellular signaling pathways in many healthy and disease states. For the first time, the present review summarizes current evidence suggesting that the beneficial properties of probiotics could be explained based on the pivotal role of miRNAs.

Video Abstract

Video Abstract

## Background

The gastrointestinal tract (GIT) is an active ecosystem within the human body, and normally contains beneficial bacteria essential for maintaining metabolism and immune cell maturation. In the gut, healthy bacteria are part of the normal microbiota that arrive in the intestine via food intake, and naturally coexist with each other. These microorganisms are in a balanced relationship with immune cells associated with the lamina propria in the gut, and lead to the stimulation and maturation of these immune cells [1].

Probiotics are defined as a group of beneficial living bacteria, which are naturally members of the intestinal microbiota, and some of them have been incorporated into food products and supplements to improve GIT health through maintaining a good microbial balance. To improve host health or to treat various infectious and non-infectious diseases, the potential properties of probiotics have been widely explored in experimental models. The mentioned beneficial effects include: a decrease in GIT inflammatory responses [2], preventive activity against malignancy [3–5], protective role against infections [6–8], allergy prevention [9, 10], inhibition of *Helicobacter pylori* growth [11], and relief of irritable bowel syndrome [12]. In addition, the antioxidant, anti-inflammatory and anticancer activity of probiotics may be explained by saturated fatty acid production in the gut [13, 14]. Although probiotics have shown encouraging results in several human diseases, such as irritable bowel syndrome, multi-drug resistant pathogens, and diabetes [15–17], more extensive studies are still needed to confirm their preliminary effectiveness on nutrition, human health, and modulation of various diseases.

MicroRNAs (miRNAs) are members of the family of non-coding RNAs, and are 19–25, nucleotides in length [18]. miRNAs are generated from endogenous primary miRNA precursors [19]. Recently, interest in miRNAs has taken off due to their roles in the treatment and development of a wide variety of diseases. Additionally, miRNAs play key roles in numerous normal physiological networks. Deregulation of miRNAs has been implicated in the pathogenesis of several disorders, including cancer and infections [20, 21].

This review summarizes the available data on the modulatory effects of probiotics on miRNA expression and function in pathological conditions,

### Probiotics in health and disease

Microbial populations found within the GIT, have numerous roles in different aspects of human health [22]. Any change in the normal microbiome can be the cause of various diseases, including gastrointestinal cancers and obesity [23, 24]. Probiotics are externally administered living microbes that are beneficial for human health via their regulatory function on the host GI microbiome, host immune system, and systemic inflammation [25–27]. Probiotics, if administered in a sufficient amount, can improve diseases, or the lessen the complications of various disorders, including GI cancers, inflammatory bowel disease (IBD), rheumatoid arthritis, obesity, and diabetes (Table 1) [106, 107]. *Lactobacillus* and *Bifidobacterium* species are the major bacterial strains that are generally consumed as probiotics [107].

**Table 1:** Selected probiotic preparations that have been investigated to treat various diseases

Physiology or pathological condition	Probiotic agent	Probiotic concentration	Duration of study	Effect (s)	Model	Sample (n)	Ref
Colorectal cancer	*Lactobacillus acidophilus, L. rhamnosus*	2 × 10^9^ CFU (colony-forming units)	12 weeks	Enhance bowel signs and QOL	Human	28	[28]
Gastric cancer	*Bifidobacterium infantis*, *Lactobacillus acidophilus*, *Enterococcus faecalis, Bacillus cereus*	> 10^6^ CFU/tablet	6–7 days	Improve immune response and decrease severity of inflammation	Human	50	[29]
Colorectal cancer	*Lactobacillus acidophilus, L. casei, L. lactis,* *Bifidobacterium bifidum, B. longum, B. infantis*	3 × 10^10^ CFU	8 weeks	Improve QOL, decrease inflammatory biomarkers, reduce side effects of chemotherapy	Human	70	[30]
Colorectal cancer	*Lactobacillus acidophilus, L. casei. L. lactis, Bifidobacterium bifidum, B. longum, B. infantis*	3 × 10^10^ CFU	1 week	Accelerate return of normal gut function	Human	20	[31]
Asthma	*Lactobacillus rhamnosus GG* (LGG)	1 × 10^10^ CFU	6 months	Enhance T-regulatory induction	Human	10	[32]
Eczema and Asthma	*Lactobacillus rhamnosus* GG (LGG)	1 × 10^10^ CFU	6 months	Prevent the eczema or asthma development	Human	92	[33]
Seasonal allergic rhinitis and Intermittent asthma	*Bifidobacterium spp* (*B. longum* BB536, *B. infantis* M-63, *B. breve* M-16V)	3 × 10^9^ CFU, 1 × 10^9^ CFU and 1 × 10^9^ CFU	4 weeks	Enhance AR and QOL	Human	40	[34]
Atopic	*Bifidobacterium bifidum*W23*, Bifi-dobacterium lactis*W52 and *Lactococcus Lactis* W58	3 × 10^9^ CFU	6 years	Minor impact on gut microbiota composition	Human	99	[35]
Necrotizing enterocolitis (NEC)	*Bifidobacterium* and *Lactobacillus*	3 × 10^9^ CFU	At least 10 days	Decreased frequency of necrotizing enterocolitis in preterm neonates	Human	52	[36]
Necrotizing enterocolitis (NEC)	*Bifidobacterium lactis* and *Lactobacillus rhamnosus*	1 × 10^8^ CFU and 1 × 10^9^ CFU	3 years	No significant effects	Human	332	[37]
Necrotizing enterocolitis (NEC)	*Lactobacillus acidophilus and Bifidobacterium bifidum*	1 × 10^9^CFU and 1 × 10^9^ CFU	8 month	No significant effects	Human	31	[38]
Inflammatory bowel disease (IBD)	*Lactobacillus rhamnosus* GR-1 and *Lactobacillus reuteri* RC-14	1 × 10^3^ CFU and 2 × 10^7^ CFU	30 days	Anti-inflammatory effects	Human	8	[39]
Inflammatory bowel disease (IBD)	*Lactobacillus acidophilus* La-5 and *Bifidobacterium* BB-12	1 × 10^6^ CFU	8 weeks	Enhanced intestinal function	Human	105	[40]
Rheumatoid arthritis (RA)	*Lactobacillus rhamnosus* GR-1 and *Lactobacillus reuteri* RC-14	2 × 10^9^ CFU	4 month	No significant effects	Human	14	[41]
Rheumatoid arthritis	*Lactobacillus acidophilus*, *L. casei* and *Bifidobacterium bifidum*	6 × 10^9^ CFU	8 weeks	Therapeutic effects	Human	30	[42]
Rheumatoid arthritis	*Lactobacillus casei* 01	1 × 10^8^ CFU	8 weeks	Reduced disease activity and inflammatory status	Human	22	[43]
Atopic dermatitis	*Lactobacillus rhamnosus* (LR)	NA	8 weeks	Reduce signs of atopic dermatitis	Human	30	[44]
Atopic dermatitis	*Lactobacillus plantarum* IS-10506	1 × 10^10^ CFU	12 weeks	Therapeutic effects	Human	12	[45]
Atopic dermatitis (AD)	*Lactobacillus rhamnosus GG* (LGG), *Bifidobacterium animalis* subsp. *lactis Bb-12* (Bb-12), d *L. acidophilus La-5* (La-5)	5 × 10^10^ CFU,5 × 10^10^ CFU and 5 × 10^9^ CFU	2 years	Decreased proportion of Th22 cells.	Human	68	[46]
Irritable bowel syndrome (IBS)	*Bacillus subtilis*, *Bifidobacterium* spp., *Lactobacillus* spp., *L. lactis*, and *Streptococcus thermophilus*	8 × 10^9^ CFU	16 weeks	Significantly improvement in IBS symptoms well tolerance	Human	181	[47]
Irritable bowel syndrome (IBS)	*Lactobacillus acidophilus* CL1285, *L. casei* LBC80R, *L. rhamnosus* CLR2	5 × 10^10^ CFU	12 weeks	Improved stool consistency and frequency, QOL, and IBS symptoms	Human	76	[48]
Irritable bowel syndrome (IBS)	*Bifidobacterium longum* (BL)	1 × 10^10^ CFU	6 weeks	Decreased depression	Human	18	[49]
Irritable bowel syndrome (IBS)	*Lactobacillus brevis* KB290	1 × 10^9^ CFU	12 weeks	Improved symptoms and inflammatory status	Human	20	[50]
Gastroenteritis	*Lactobacillus rhamnosus* GG	1 × 10^10^ CFU	5 days	No significant effects	Human	468	[51]
Acute gastroenteritis (AGE)	*Lactobacillus rhamnosus* (LGG)	1 × 10^10^ CFU (twice a day)	5 days	-	Human	-	[52]
Gastroenteritis	*Lactobacillus rhamnosus* GG (LGG)	1 × 10^10^ CFU	4 weeks	Immuno-modulatory effects	Human	65	[53]
Eczema	*Lactobacillus salivarius* CUL61, *L. paracasei* CUL08, *Bifidobacterium animalis* subspecies *lactis* CUL34 and *B. bifidum* CUL20	1 × 10^10^ CFU	2 years	Prevent eczema	Human	187	[54]
Eczema	*Bifidobacterium longum* (BL999) and *Lactobacillus rhamnosus* (LPR)	NA	5 years	No significant effects	Human	124	[55]
Allergic rhinitis	*Bifidobacterium lactis* NCC 2818	4 × 10^9^ CFU	8 weeks	Improved immune parameters and allergic symptoms	Human	10	[56]
Allergic rhinitis	*Lactobacillus paracasei* LP-33	2 × 10^9^ CFU	5 weeks	Enhanced QOL	Human	179	[57]
Allergic rhinitis	*Lactobacillus paracasei* HF.A00232 (LP)	5 × 10^9^ CFU	8 weeks	No significant effects	Human	32	[58]
Celiac disease	*Bifidobacterium longum* CECT 7347	1 × 10^9^ CFU	3 months	Enhanced health status	Human	17	[59]
Celiac disease	*Bifidobacterium infantis*	2 × 10^9^ CFU	3 weeks	-	Human	12	[60]
Celiac disease	*Bifidobacterium breve* BR03 and B632	1 × 10^9^ CFU	3 month	Reduced TNF-α levels	Human	22	[61]
Obesity	*Lactobacillus casei* strain *Shirota* (LcS)	≥ 4 × 10^10^ CFU	6 months	Decreased body weight and increased high density lipoprotein cholesterol concentration	Human	12	[62]
Obesity	*Lactobacillus rhamnosus* (LPR)	1.6 × 10^8^ CFU	12 weeks	Improve fasting fullness and cognitive restraint in men	Human	62	[63]
Obesity	*Lactobacillus curvatus* HY7601, *L. plantarum* KY1032	5 × 10^9^ CFU	12 weeks	Induced weight loss and decreased adiposity .	Human	32	[64]
Type I diabetes and Type II diabetes	*Lactobacillus acidophilus* ZT-L1, *Bifidobacterium bifidum* ZT-B1, *L.* *reuteri* ZT-Lre, *L. fermentum* ZT-L3	8 × 10^9^ CFU	12 weeks	Beneficial effects on glycemic control and markers of cardio-metabolic risk.	Human	30	[65]
Type II diabetes	*Bifidobacterium bifidum* W23, *B. lactis* W52, *L. acidophilus* W37, *L.* *brevis* W63, *L. casei* W56, *L. salivarius* W24, *Lactococcus lactis* W19 and *L.s lactis* W58	2.5 × 10^9^ CFU	12 weeks	Enhanced HOMA-IR	Human	39	[66]
Type II diabetes	*Lactobacillus reuteri* DSM 17938	1 × 10^8^ CFU	12 weeks	No significant effects	Human	30	[67]
Type II diabetes	concentrated biomass of 14 probiotic bacteria genera *Bifidobacterium, Lactobacillus, Lactococcus, Propionibacterium*	*Lactobacillus* + *Lactococcus* (6 × 10^10^ CFU/g), *Bifidobacterium* (1 × 10^10^/g), *Propionibacterium* (3 × 10^10^/g) and *Acetobacter* (1 × 10^6^/g)	8 weeks	Reversed insulin resistance	Human	31	[68]
Type II diabetes	*Lactobacillus acidophilus* La-5 and *Bifidobacterium animalis* subsp *lactis* BB-12	1 × 10^9^ CFU	6 weeks	Enhanced glycemic control in T2D patients, however, the intake of fermented milk seems to be involved with other metabolic changes, such as reduced inflammatory cytokines (TNF-α and resistin) .	Human	23	[69]
HIV	*Lactobacillus casei Shirota* (LcS)	6.5 × 10^8^ CFU	8 weeks	Immunological and virological effects	Human	20	[70]
HIV	*Lactobacillus plantarum*, *Streptococcus thermophilus*, *Bifidobacterium* *breve*, *L. paracasei*, *L. delbrueckii* subsp*. bulgaricus*, *L. acidophilus*, *B. longum*, *B. infantis*	1.8 × 10^12^ CFU	6 months	Beneficial effects on the reconstitution of physical and immunological integrity of the mucosal intestinal barrier	Human	10	[71]
HIV	*Saccharomyces boulardii*	NA	12 weeks	Therapeutic effects	Human	22	[72]
Non-alcoholic fatty liver disease	a mixture of eight probiotic strains (*Streptococcus thermophilus, bifidobacteria* SPP*., Lactobacillus acidophilus, L. plantarum, L. paracasei, and L. delbrueckii subsp. bulgaricus)*	NA	4 month	Reduced body mass index (BMI) in probiotic supplemented children	Human	22	[73]
Non-alcoholic fatty liver disease	*Lactobacillus acidophilus* La5 and *Bifidobacterium lactis* Bb12	6.46 × 10^6^ and 4.97 × 10^6^ CFU/g	8 weeks	Improved hepatic enzymes, serum total cholesterol, and low density lipoprotein cholesterol levels	Human	36	[74]
Non-alcoholic fatty liver disease	*Lactobacillus* *casei, L. acidophilus*, *L. rhamnosus, L. bulgaricus, Bifidobacterium breve, B. longum,* and *Streptococcus* *thermophilus*.	Lactobacillus casei (3 × 10^9^ CFU/g), Lactobacillus acidophilus (3 × 10^10^ CFU/ g), Lactobacillus rhamnosus (7 × 10^9^ CFU/g), Lactobacillus bulgaricus (5 × 10^8^ CFU/g), Bifidobacterium breve (2 × 10^10^ CFU/g), Bifidobacterium longum (1 × 10^9^ CFU/g), and Streptococcus thermophilus (3 × 10^8^ CFU/g).	8 weeks	Reduced insulin requirement, reversed insulin resistance, TNF-α, and IL-6	Human	21	[75]
Non-alcoholic steatohepatitis	*Lactobacillus casei*, *L. rhamnosus*, *L. bulgaris*, *Bifidobacterium longum,* and *Streptococcus thermophilus*	1 × 10^8^ CFU	12 weeks	Reduced body mass index (BMI) and serum cholesterol	Human	38	[76]
Non-alcoholic steatohepatitis	*Lactobacillus plantarum, L. deslbrueckii, L. acidophilus, L. rhamnosus* and *Bifidobacterium bifidum*	2 × 10^8^ CFU	6 months	No changes in body mass index, waist circumference, glucose or lipid levels but decreased liver fat and aspartate transaminase (AST) level	Human	10	[77]
Urinary Tract Infections	*Lactobacillus crispatus* GAI 98322	1 × 10^8^ CFU	1 year	Prevent recurrence of UTI	Human	9	[78]
Urinary Tract Infections	*Lactobacillus crispatus*	1 × 10^8^ CFU	10 weeks	Less recurrence of UTI	Human	43	[79]
Urinary Tract Infections	*Lactobacillus GG*	6 × 10^9^ CFU	1 week	Decreased incidence of UTIs, necrotizing enterocolitis (NEC) and sepsis	Human	295	[80]
Urinary Tract Infections	*Lactobacillus crispatus CTV-05*	5 × 10^8^ CFU	5 days	Minimal side effects	Human	15	[81]
Vaccine Adjuvants	*Bifidobacterium lactis*, *Lactobacillus acidophilus*, *L. plantarum*, *L. paracasei* and *L. salivarius*	2 × 10^10^ CFU	3 weeks	Faster immune response. Specific probiotics may be adjuvants to humoral immune response following oral vaccination	Human	63	[82]
Vaccine Adjuvants	*Lactobacillus fermentum* CECT5716	1 × 10^10^ CFU	4 weeks	Potentiated the immunologic response and improved systemic protection from infection	Human	25	[83]
Vaccine Adjuvants	*Lactobacillus* GG	1 × 10^10^ CFU	4 weeks	Improved influenza vaccine immunogenicity	Human	19	[84]
Plaque and carries	*Lactobacillus reuteri*	1 × 10^8^ CFU	1 years	Decreased caries prevalence and gingivitis score	Human	60	[85]
Plaque and carries	*Streptococcus uberis* KJ2™, *Streptococcus. oralis* KJ3™*, Streptococcus. rattus* JH145	1 × 10^8^ CFU	1 year	Reduced early childhood caries development	Human	54	[86]
Plaque and carries	*Lactobacillus brevis* CD2	NA	6 weeks	Beneficial effects on variables associated with oral health	Human	91	[87]
Plaque and carries	*Bifidobacterium animalis subsp. lactis* BB-12	1 × 10^10^ CFU	2 years	No significant effects	Human	32	[88]
Plaque and carries	*Bifidobacterium animalis* subsp*. lactis* DN-173010	≥ 10^8^ CFU	4 weeks	Effect on plaque accumulation and gingival inflammatory parameters	Human	26	[89]
Oral wounds	A mixture of two probiotic strains, *Lactobacilli reuteri* DSM 17938 and ATCC PTA 5289	2 × 10^8^ CFU/mL	1 week	No significant effects	Human	-	[90]
oral lichen planus (OLP)	*Lactobacilli reuteri* (DSM 17938 and ATCC PTA 5289)	NA	16 weeks	No significant effects	Human	9	[91]
Periodontitis	*L. rhamnosus* SP1	2 × 10^7^ CFU	3 month	Clinical and microbiological improvements.	Human	16	[92]
Periodontitis	*Lactobacillus reuteri*	1 × 10^8^ CFU	3 weeks	Decreased pro-inflammatory cytokine response and improved clinical parameters	Human	24	[93]
Periodontitis	*Lactobacillus reuteri*	1 × 10^8^ CFU	12 weeks	Useful adjunct to scaling and root planing (SRP) in chronic periodontitis.	Human	15	[94]
chronic periodontitis (CP)	*Lactobacillus Reuteri*	NA	3 weeks	Reduced inflammatory markers	Human	15	[95]
Periodontitis	*L. rhamnosus* SP1	2 × 10^7^ CFU	3 months	Clinical improvement	Human	12	[96]
Gingivitis	*Bifidobacterium animalis*	≥ 10^8^ CFU	4 weeks	Effects on plaque accumulation and gingival inflammation	Human	26	[89]
Gingivitis	*Lactobacillus rhamnosus* PB01*,* DSM 14869 and *Lactobacillus curvatus* EB10*,* DSM 32307	≤ 10^8^ CFU/tablet	4 weeks	Enhanced gingival health	Human	23	[97]
Gingivitis	*Bacillus subtilis,* *Bacillus megaterium* and *Bacillus pumulu*s as a toothpaste	5 × 10^7^ CFU	8 weeks	No significant effects	Human	20	[98]
Gingivitis	*Lactobacillus plantarum*, *Lactobacillus brevis* and *Pediococcus acidilactici*	NA	6 weeks	Significant changes in mean gingival index	Human	29	[99]
Pregnancy Gingivitis	*Lactobacillus reuteri* DSM 17938	≥ 10^8^ CFU	7 weeks	Useful adjunct in the control of pregnancy gingivitis	Human	24	[100]
Halitosis	*Lactobacillus salivarius* WB21	2 × 10^9^ CFU	4 weeks	Improved halitosis	Human	20	[101]
Halitosis	*Lactobacillus brevis* CD2	NA	2 weeks	No significant effects	Human	10	[102]
Candida infection	*Lactobacillus reuteri*	1 × 10^8^ CFU	1 month	Decreased incidence of sepsis in addition to improving symptoms and feeding	Human	150	[103]
Candida infection	*Lactobacillus fermentum* LF10 and *L.* *acidophilus* LA02	≥ 4 × 10^8^ CFU	11 weeks	-	Human	57	[104]
Candida infection	*Lactobacillus acidophilus, L.* *rhamnosus, Streptococcus thermophilus*, and *L. delbrueckii* *subsp. bulgaricus*	NA		Prevented relapse	Human	201	[105]

**Fig 1:**
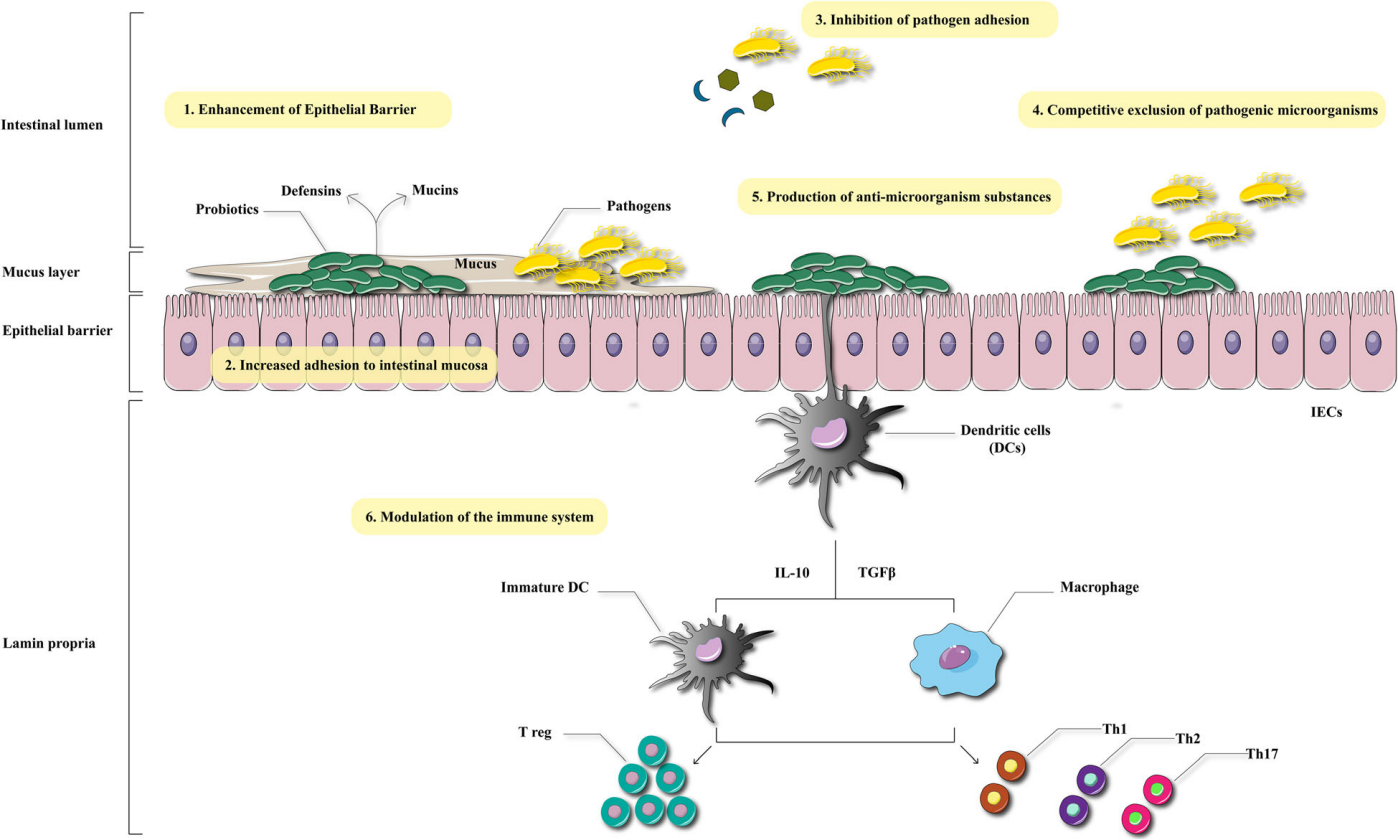
Major mechanisms of action of probiotics. Probiotics exert its therapeutic effects via different mechanisms including enhancement of epithelial barrier, inhibition of pathogen adhesion, and modulation of immune system. This figure adapted from  Bermudez-Brito  et al (Ann Nutr Metab 2012;61:160–174)

The major mechanisms of action of probiotics, include enhancement of the gut epithelial barrier, adhesion to intestinal mucosa, and concomitant inhibition of pathogen adhesion, competitive exclusion of pathogenic microorganisms, production of anti-microbial substances, and modulation of the immune system (Fig. 1).

Epidemiological studies clearly show that, despite all the advances made in the field of diagnosis, prevention and treatment of cancer, the prevalence of cancer is still increasing. About 80% of all types of cancer are due to environmental and lifestyle factors [108]. Due to the high burden of cancer around the world, effective treatment cancer is very important [109]. There are many treatment methods such as chemotherapy, radiotherapy, targeted therapy and immunotherapy, but their overall efficacy is still not satisfactory [110, 111]. Probiotics have recently been used to improve cancer treatment, relieve symptoms and increase the quality of life [112]. One of the reasons for the occurrence of cancers, in particular GIT cancers, may be changes in the normal GI microbial flora, and therefore the use probiotics is attractive to modulate these changes, reduce complications, or even treat cancer [113]. In one study by Gao et al., probiotic therapy reduced the number of mucosal-associated pathogens in patients with colorectal cancer (CRC) by altering the profile of microbial flora in the mucosa. A total of 22 CRC patients completed the trial. Patients were randomized into a PGT group (*n* = 11) taking probiotics or CGT group (*n* = 11) taking preoperative placebo. It should be noted that the participants in the PGT group took an encapsulated probiotic combination consisting of living *Bifidobacterium longum*, *Enterococcus faecalis*, and *Lactobacillus acidophilus* (1:1:1) with at least 1×10^7^ CFU/g viable cells, 3 times a day, with a total daily dosage of 6×10^7^ CFU for 5 days. The participants in the CGT group took only encapsulated maltodextrin 3 times a day. The results showed that the probiotic supplement regimen could efficiently modify the composition and diversity of the gut microflora. It could also suppress specific potential pathogens such as *Peptostreptococcus* and *Fusobacterium* strains. In addition, probiotics enhanced the numbers of specific beneficial microorganisms [114]. Abnormal blood vessels and hypoxic and necrotic regions are common features of solid tumors and related to the malignant phenotype and therapy resistance. Certain obligate or facultative anaerobic bacteria exhibit inherent ability to colonize and proliferate within solid tumors in vivo. Escherichia coli Nissle 1917, a non-pathogenic probiotic in European markets, has been known to proliferate selectively in the interface between the viable and necrotic regions of solid tumors. Li et al. established a tumor-targeting therapy system using the genetically engineered E. coli Nissle 1917 for targeted delivery of cytotoxic compounds, including glidobactin, colibactin, and luminmide. Biosynthetic gene clusters of these cytotoxic compounds were introduced into E. coli Nissle 1917 and the corresponding compounds were detected in the resultant recombinant strains. The recombinant E. coli Nissle 1917 showed cytotoxic activity in vitro and in vivo as well, and suppressed the tumor growth [115]. Another study was conducted to evaluate the effect of probiotic supplementation in patients with laryngeal cancer. After the intervention, biochemical markers of stress were reduced. A total of 20 healthy controls and 30 patients with laryngeal cancer were included. Then, for two weeks prior to the surgical operation, 20 patients were randomly assigned to take a placebo or probiotic supplement (Clostridium butyricum; 420 mg/capsule) two times per day. In addition, the degree of anxiety and the heart rate were assessed. It was found that the level of serum corticotropin-releasing factor (CRF) in patients with laryngeal cancer was increased as they approached the time of surgery, but no corresponding increase in CRF, anxiety or heart rate was seen after probiotic use. Probiotics reduced the level of the patient anxiety on the Hamilton Anxiety Scale (HAMA) from 19.8 to 10.2. Consequently, clinical anxiety and biochemical features of stress were decreased by probiotics administration in the participants assigned for laryngectomy [116]. In another study carried out on patients with gastric cancer, the results showed that the combination of dietary fiber and probiotics was effective in treating post-operative diarrhea. The study included 120 patients suffering from GC. Patients were assigned to one of 3 groups as follows: (1) Fiber-enriched nutrition formula (FE group, *n* =  40), (2) Fiber-free nutrition formula (FF group, n  =  40), and (3) Fiber and probiotic-enriched nutrition formula, a combination of live bifidobacterium and lactobacillus in tablets , (FEP group, n  =  40). Then, each patient received enteral nutrition (EN) formulae for seven successive days after the surgical operation [117]. Earlier investigations had shown that addition of the fiber or probiotics may preserve the intestinal microecology, and diminish diarrhea related to EN [118]. Dietary fiber is a kind of carbohydrate polymer, which cannot be digested by humans. Some researchers have shown that the effects of combined probiotics and fiber on the treatment of diarrhea were indecisive [119–121]. According to the present RCT investigation, the combination of probiotics and fiber may reduce diarrhea, augment intestinal motility, and diminish intestinal dysfunction in the post-operative GC patients receiving EN. Additionally, probiotics may shorten the length of hospital stay (LOHS) as part of enhanced recovery after surgery (ERAS) protocols. Therefore, the combination of fiber and probiotics when beginning EN, may avoid diarrhea related to EN, improving comfort, and enhancing recovery after surgical operation [117].

Ventilator-associated pneumonia (VAP) is often caused by aspiration of pathogenic bacteria from the oropharynx. Oral decontamination by using antiseptics, such as antibiotics or chlorhexidine (CHX), can be used as prophylaxis. Klarin et al. examined the probiotic effect of bacteria Lactobacillus plantarum 299 (Lp299) as CHX in reducing the pathogenic bacteria in the oropharynx. Fifty tracheally intubated, mechanically ventilated, critically ill patients critically ill patients administrated to either oral cleansing by 0.1% CHX solution or to the same washing procedure and oral using of an emulsion of Lp299. Oropharynx samples showed that pathogenic bacteria that were not present at inclusion were detected in the patients treated with Lp299 was less than those in control group [122].

Inflammatory bowel disease (IBD) consists of two disorders, Crohn's disease and ulcerative colitis [123]. In the pathogenesis of IBD, it is thought that pathogenic or resident luminal bacteria continuously activate the mucosal and systemic immune systems, and ultimately cause an inflammatory cascade [124]. IBD is a chronic immunological disease that is related to lack of dietary fiber, saturated fatty acids, poor sleep, and low levels of vitamin D in the body [123]. For medical therapy, drugs such as immunomodulators and 5-aminosalicylic acid (5-ASA) can be used [125]. Antibiotics and probiotics are also used to treat IBD [126]. So far, several studies have been performed to evaluate the efficacy of probiotics in IBD. In one study carried out by Shadnoush et al., supplementation with probiotics improved intestinal function in patients with IBD. A total of 305 participants were classified into 3 groups. Group A (IBD patients taking probiotic yogurt (contained Lactobacillus acidophilus La-5 and Bifidobacterium BB-12): *n* = 105), group B (IBD patients taking a placebo: *n* = 105), or control group (healthy persons taking probiotic yogurt: *n* = 95). Stool samples were obtained before and after eight weeks of intervention. Afterwards, the numbers of *Bifidobacterium*, *Lactobacillus*, and *Bacteroides* species in the stool samples were measured. It was found that the mean number of *Bifidobacterium*, *Lactobacillus*, and *Bacteroides* CFU in group A was increased compared to group B. Moreover, the mean number of all 3 bacteria was significantly different between groups A and B compared to healthy control group C. The differences between the two groups were seen both at the base-line and the completion of the study. It has been found that consuming probiotic yogurt by IBD patients can contribute to the improved intestinal function via enhancing the numbers of the beneficial bacteria in the gut. Nonetheless, it is still necessary to do more studies to confirm this concept [40]. In one study that used a *Lactobacillus reuteri* rectal infusion in children with chronic ulcerative colitis (UC), mucosal inflammation and the expression of some pro-inflammatory cytokines was decreased. Oliva et al. investigated the effects of a *Lactobacillus* (L) *reuteri* ATCC 55730 enema on children with active distal UC, and measured inflammation and cytokine expression in the rectal mucosa. In a prospective, randomized, placebo-controlled trial, in addition to taking oral mesalazine, the patients (n=40) received an enema solution containing 10^10^ CFU of *L. reuteri* or placebo for eight weeks. The Mayo score (endoscopic and clinical characteristics) was considerably reduced in the *L. reuteri* group in comparison to the placebo. Moreover, histological scores showed a considerable decline in the *L. reuteri* group. In addition, at the post-trial assessment of the level of mucosal cytokine expression, the anti-inflammatory IL-10 was significantly increased, while the pro-inflammatory TNFα, IL-8 and IL-1β were reduced in the *L. reuteri* group [127].

Kekkonen eta l. investigated production of cytokine in human peripheral blood mononuclear cells (PBMC) in response to stimulation with probiotic bacteria including *Streptococcus thermophilus* THS, *Lactobacillus rhamnosus* GG (ATCC 53103), *Lactobacillus rhamnosus* Lc705 (DSM 7061), *Lactobacillus helveticus* 1129, *Lactobacillus helveticus* Lb 161, *Bifidobacterium longum* 1/10, *Bifidobacterium animalis* ssp. *lactis* Bb12, *Bifidobacterium breve* Bb99 (DSM 13692), *Lactococcus lactis* ssp. *cremoris* ARH74 (DSM 18891), *Leuconostoc mesenteroides* ssp. *cremoris* PIA2 (DSM 18892) and *Propionibacterium freudenreichii* ssp. *shermanii* JS (DSM 7067). All of examined bacteria could induce TNF-α production. Streptococcus and Leuconostoc induced Th1 type cytokines IL-12 and IFN-γ more than other. All Propionibacterium and Bifidobacterium strains induced higher IL-10 production. They showed that Leuconostoc mesenteroides ssp. cremoris and Streptococcus thermophilus are more potent inducers of Th1 type cytokines IL-12 and IFN-γ than the probiotic Lactobacillus strains [128].

Atopic dermatitis (AD) is an inflammatory skin disease can be due to the imbalance between Tcell-related immune responses. Sheikhi et al. investigated the effects of lactobacillus Bulgaricus in the yogurt culture on the secretion of Th1/Th2/Treg type cytokines by PBMCsin 20 children with AD. Results showed that L. Delbrueckii subsp. Bulgaricus significantly up-regulated the secretion of IL-10, IL-12 and IFN-γ, while secretion of IL-4 was decreased by PBMCs compared to control [129].

Some studies have suggested that arthralgia is a common extra-intestinal manifestation of IBD. It is possible that disturbing the immune profile within the gut plays a role in the pathogenesis of arthralgia. A study by Karimi showed that administration of probiotics (VSL#3) could improve pouchitis in IBD patients. The safety and efficacy of VSL#3 administration for 2 weeks in patients with quiescent IBD also suffering from arthralgia, was assessed in an open-label trial. The pre-treatment and post-treatment intensity of joint pain was measured using a visual analog scale and the Ritchie Articular Index. Moreover the Truelove-Witts and the Harvey-Bradshaw scores were used to assess severity of IBD symptoms. 16 of 29 patients completed the trial. 10 of these 16 patients showed a remarkable improvement in joint pain using the Ritchie Articular Index. No patients suffered a relapse of intestinal disease while on probiotics. The above results indicated that the probiotic supplement VSL#3 could be a good therapeutic option for arthralgia inpatients suffering from IBD. Since probiotics can also serve as an IBD treatment, patients suffering from comcomitant arthralgia could take advantage of a dual treatment modality [130]. Another study carried out in laboratory dogs suggested that the anti-inflammatory effects of probiotic administration could be due to reduced mucosal immune cell infiltration, accompanied by increased levels of putrescine (PUT), ornithine decarboxylase (ODC) and diamino-oxidase (DAO), which play an anti-inflammatory role [131].

Irritable bowel syndrome (IBS) is a common chronic disease of the GIT with an incidence of 3 - 20 % in the US [132, 133]. The exact mechanisms of IBS pathogenesis are still not fully understood, but immunological disturbances and low levels of inflammation contribute to the symptoms of the disease [134]. IBS is a complex of different symptoms, such as abdominal pain, diarrhea, constipation, and general bodily weakness [132]. The main treatments are drug therapy with anti-spasmodic and anti-diarrheal drugs, fiber-rich diet for constipation, and supportive treatment with low dose antidepressants [132]. In addition to these treatments, probiotics can also be used to treat IBS; Nevertheless the role of probiotic microorganisms in the treatment of IBS has not yet been fully confirmed [135]. Several studies have shown the reduction of IBS symptoms by probiotic administration; however, there is still not enough evidence about their impact on psychiatric comorbidity. For example, Pinto-Sanchez et al. carried out a prospective study to evaluate the effects of *Bifidobacterium longum* NCC3001 (BL) on depression and anxiety symptoms in patients suffering from IBS. The researchers randomly selected 44 IBS patients suffering from diarrhea for the trial. The patients took either BL (n=22) or placebo (n=22) for 6 weeks. Afterwards, the levels of depression and anxiety, IBS symptoms, quality of life (QOL), and somatization were measured at weeks 0, 6, and 10. This study demonstrated a reduction in depression by probiotic BL; however, they observed no reduction in the anxiety scores. Moreover, the probiotic BL increased QOL in IBS patients. They found a correlation between the psychological improvements and changes in the brain activation pattern, indicating a reduction in limbic reactivity by probiotics [49]. Mezzasalma et al. investigated the efficacy of a supplementary regimen containing multi-species probiotics to alleviate patient IBS symptoms, such as constipation (IBS-C), and also measured their gut microbiota. They conducted their study in 150 IBS-C participants who received orally administered probiotic mixtures F_1 or F_2 or else placebo F_3 for 60 days. The results showed that the improvement in symptoms was greater in the probiotic group in comparison with the placebo group. The symptoms also remained in remission during the follow-up period. Moreover, fecal analyses showed that the probiotics enhanced fecal bacterial DNA in participants who received F_1 and F_2, but not with F_3. A similar level continued during the follow-up course [136]. In another study, Choi et al. explored the effectiveness of a combined treatment with mosapride and probiotics in IBS patients without diarrhea. They randomly assigned 285 IBS patients to receive over 4 weeks, a combined treatment with probiotics (*Streptococcus faecium* & *Bacillus subtilis*) plus mosapride at 4 different dosages (groups 1-4), or a placebo. The proportion gaining AR at the fourth week was greater in all treatment groups in comparison with the placebo group. Moreover, the proportion of the patients who improved on the SGA was also greater in the treatment groups compared to the placebo group. In addition, abdominal discomfort and pain scores in treatment group 4 showed the best improvement in comparison to the placebo group. In patients suffering from constipation-predominant IBS, greater improvement was observed in stool frequency and consistency in the treatment groups 4 and 1 as compared to the placebo group [137].

Celiac disease (CD) is a chronic immune-mediated disease caused by the consumption of foods containing gluten, especially wheat, and is most often seen in genetically-predisposed individuals [138]. The part of the intestines involved in CD is the proximal section of the small intestine [139]. The prevalence of celiac disease is different worldwide, but its incidence has increased in the last few decades [140]. Currently, the only effective treatment for patients with CD is a gluten-free diet (GFD) [141]. Of course, the effectiveness of this treatment depends on the strict avoidance of gluten, which may sometimes be difficult [141]. In patients with celiac disease, as compared to healthy people, the beneficial gut microbes have been shown to be decreased, and the potentially pathogenic microbes are increased [142]. This change in the microbiome increases the inflammatory response in the intestine and worsens celiac disease [142]. Considering the role of probiotics in modulating microbial populations, probiotics could be used to reduce inflammatory response and to improve the symptoms of celiac disease. In a study evaluating the effects of *Bifidobacterium infantis* Natren Life Start (NLS) strain super strain in patients with CD, it was concluded that this strain could relieve the symptoms of untreated individuals. 22 patients who were positive for two different CD-specific tests were enrolled in this study. The patients were randomly assigned to take two capsules before meals for three weeks, containing either placebo or *Bifidobacterium infantis* super strain (Lifestart 2). It was found that *B. infantis* alleviated the symptoms in the untreated CD patients. Moreover, probiotics produced a number of immunologic alterations, but the abnormal intestinal permeability was not affected [60]. Another study conducted by Olivares et al. used *Bifidobacterium longum CECT 7347* in children with newly diagnosed CD, showed that this probiotic could improve the QOL in subjects. They assessed the effects of oral administration of the *B. longum* CECT 7347 in 33 children who were diagnosed with CD, after they had been on a gluten-free diet (GFD) for a three-month period. It was concluded that oral administration of *B. longum* CECT 7347 in combination with a GFD decreased the potentially pro-inflammatory bacteria in the gut (*B. fragilis* group) which have been associated with CD in earlier studies, as well as fecal sIgA (soluble immunoglobulin A). In addition, *B. longum* CECT 7347 decreased activated T-lymphocytes and inflammatory markers (TNF-a), possibly showing improved immune homeostasis in CD patients [59]. Another study that investigated the effects of a probiotic consisting of two strains *Bifidobacterium breve BR03* and *B. breve B632* in children with CD combined with a GFD, showed that the TNF-α inflammatory cytokine was decreased in these children after the intervention [143]. *In vitro* investigations also showed that several strains of *Bifidobacteria* could lower levels of pro-inflammatory cytokines (TNF-α, IFN-γ, & IL-2) and increase levels of the anti-inflammatory cytokine (IL-10). *Bifidobacteria* may be able to reverse the pro-inflammatory milieu caused by the microbiota of patients with CD [144–146]. Moreover, many researchers have shown that *B. breve* strains can exert immuno-modulatory effects both *in vitro* and *in vivo* [147, 148]. It has been established that these probiotics bacteria possess the Qualified Presumption of Safety status [149]. Two recent studies have shown that intestinal inflammation can be prevented by *B. breve* strains via induction of a population of T-regulatory 1 (Tr1) cells that secrete IL-10 [150, 151]. It is possible that gliadin-specific Tr1 cell clones could suppress the proliferation of pathogenic T-cells in CD patients [152]. Autoimmune-type dysfunction in known to be increased in CD patients [153]. Supplementation with *B. breve* probiotics plus a GFD can ameliorate the pro-inflammatory environment in the CD gut, and thus reduce the recurrence of the disease. In another study, Klemenak et al. determined the effects of a combined treatment with *B. breve* strains B632 and BR03 plus a GFD on the immunological function of CD children by measuring serum levels of TNF-a and IL-10. They randomly assigned 49 CD children on a GFD into treatment and placebo groups, plus 18 healthy children in a control group. The first group (24 CD children) took *B. breve* strains BR03 and B632 (2×10^9^ colony-forming units) per day, while the second group (25 CD children) took a placebo for three months. The results showed a significant reduction in the level of TNF-α in the first group after the administration of *B. breve* for three months. Follow-up three months after the end of the study showed that level of TNF-α increased once again. The levels of IL-10 were below the detection level in each group. They concluded that probiotic *B. breve* strains could decrease the proinflammatory cytokine TNF-a in CD children on a GFD [61]. Quagliariello et al. also found that a supplement of *Bifidobacterium breve* (*B632 and BR03*) in CD children treated with GFD could increase the amount of beneficial microbial compounds in the gut [154].

In a study the response of intestinal epithelial cells to Enterococcus faecium NCIMB 10415 (E. faecium) and two pathogenic E. coli strains was examined, with focus on the probiotic modulation of the response to the pathogenic challenge. Intestinal cells (IPEC-J2 and Caco-2) were incubated without bacteria (control), with E. faecium, with enteropathogenic (EPEC) or enterotoxigenic E. coli (ETEC) each alone or in combination with E. faecium. Results showed that the ETEC strain decreased transepithelial resistance (TER) and increased IL-8 mRNA and protein expression in both cell lines compared with control cells, an effect that could be prevented by pre- and coincubation with E. faecium. Similar effects were observed for the increased expression of heat shock protein 70 in Caco-2 cells. When the cells were challenged by the EPEC strain, no such pattern of changes could be observed. The reduced decrease in TER and the reduction of the proinflammatory and stress response of enterocytes following pathogenic challenge indicate the protective effect of the probiotic [155].

Rheumatoid arthritis (RA) is the most common type of inflammatory arthritis, and is a main cause of disability [156]. Inflammatory cytokines play an important role in the pathogenesis of RA. An imbalance between pro-inflammatory and anti-inflammatory cytokines causes induction of autoimmunity and chronic inflammation resulting in joint damage [157]. Epidemiological studies have estimated a 0.5-1% prevalence worldwide, and an annual average incidence of 0.02-0.05% in the Northern European and North American regions [158]. There are several drugs given to patients with RA, one of which is methotrexate (MTX) [159]. However all of these drugs have side effects that can adversely affect the QOL of RA patients. For example, MTX commonly causes GI symptoms and elevation of hepatic enzymes, while severe hemocytopenia and MTX pneumonitis occur less frequently [160]. Given the role that probiotics play in down-regulating inflammatory cytokines [161], they could be substituted for drugs in the treatment of RA. In a study by Mandel, supplementation with *Bacillus coagulans GBI-30* was shown to be effective in patients with RA [162]. A probiotic mixture of three strains of *Lactobacillus acidophilus*, *Lactobacillus casei* and *Bifidobacterium bifidum* was given for 8 weeks to patients with RA. Probiotics showed positive effects on serum insulin levels, assessment of B cell function (HOMA-B), and serum high-sensitivity C-reactive protein (hs-CRP) concentrations in a study by Zamani et al [42]. Another study was conducted to investigate the effects of *Lactobacillus casei 01* in RA patients. At the end of the intervention it was found that this strain could reduce the symptoms of this disease. The researchers randomly assigned women with established RA to receive either a placebo or one capsule containing 10^8^ colony-forming units (CFU) of *L. casei* 01 for eight weeks. Serum levels of cytokines IL-1β, IL-10, IL-6, TNF-α and IL-12 were measured. They concluded that *L. casei* 01 could decrease the serum level of hs-CRP, improve swollen joints and tenderness, and increase the global health score and disease activity score-28 (P < 0.05). They showed a significant difference between both groups for IL-12, IL-10, and TNF-α levels over the study (P<0.05), in favor of the probiotic group. There were no adverse effects in the intervention group. They concluded that probiotics could be a useful supplement for patients with RA, to alleviate symptoms and reduce inflammatory cytokines [163]. In a study by Hatakka et al. supplementation with *Lactobacillus rhamnosus GG (LGG)* reduced the severity of RA. However, probiotic therapy with *Lactobacillus* GG showed no effects on RA severity. Therefore, it is necessary to do more research into probiotic bacteria for RA patients [164].

### Modulation of microRNA by probiotics

**Fig 2:**
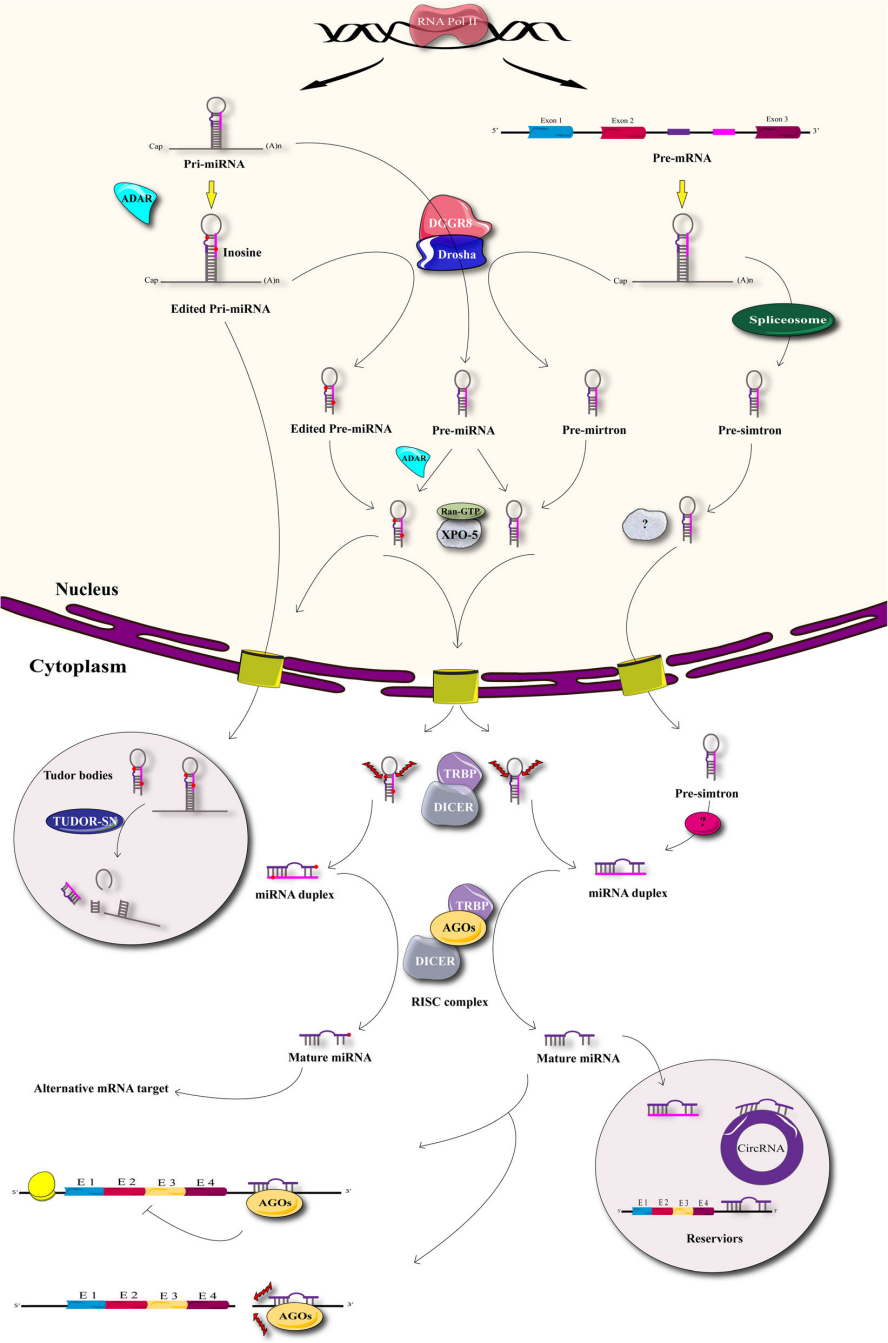
Biogenesis of miRNA. Starts in the nucleus when miRNA genes are transcribed by RNA polymerase II as large polyadenylated RNA molecules named primary miRNAs (pri-miRNAs). Pri-miRNAs are processed in the nucleus by RNase III Drosha and microprocessor complex subunit DGCR8. As a result, pri-miRNAs are cleaved into smaller double-stranded RNA (dsRNA) molecules known as pre-miRNAs and then are exported to the cytoplasm by exportin 5 (XPO5). Pre-miRNAs in the cytoplasm are cleaved and despoiled of their loops by the RNase III enzyme Dicer in association with TRBP into mature miRNAs consisting of a ∼22-nucleotide duplex. The last processing step is carried out by a ribonucleoprotein complex known as RNA-induced silencing complex (RISC), which can unwind both strands. Although either strand of the miRNA duplex could potentially act as a mature miRNA, usually only one of the strands is incorporated into the RISC complex to induce mRNA silencing. RISC-loading complex (RLC), consisted of Dicermm, Argonaute 2 (Ago2), and TRBP. Once loaded, the RISC complex finds a complementary strand, activates RNase and cleaves the RNA

MicroRNAs (miRNAs) are short non-coding RNAs comprising 20 to 24 nucleotide bases, which play important roles in all the biological and physiological pathways in multicellular organisms [165]**.** Figure 2 shows the scheme of miRNA biogenesis. Dysregulation of microRNAs plays an important role in the pathogenesis of many different diseases [165]. These diseases include, central nervous system disorders [166], autoimmune diseases [167], cancers [168] and many other common diseases worldwide. Some treatments that are used to cure these diseases act by influencing gene expression and affecting miRNA regulation [169–171].

Probiotics are among other biological factors that have recently been discussed with regard to whether they have any effects on miRNAs. Many laboratory studies have so far been done to investigate the effects of probiotics on miRNAs (Table 2).

**Table 2:** Modulation of microRNA by probiotics in pathological conditions

MicroRNA	Probiotics	Probiotic concentrations	Expression	Target gene	Effects	Model	Sample (n)	Ref
miR-215-5p, miR-10b-5p, miR-21-5p, miR-26a-5p, miR-22-3p, miR-10a-5p, miR-148a-3p, miR-194, miR-92-3p, miR-30d, miR-181a-5p, miR-429-3p, let-7f-5p, miR-30a-5p, miR-133a-3p, miR-199-3p, miR-30c-5p, miR-200a-3p, miR-126-5p, miR-27b-3p	*Lactobacillus plantarum* Z01 (LPZ01)	1 × 10^8^ CFU/mL	Down-regulation of miR-215-5p, miR-3525, miR-122-5p and up regulation of miR-193a-5p, miR-375 and miR-215-5p	cAMP-dependent protein kinase activity, stress-activated MAPK cascade, MAPK and Wnt signaling pathways.	Decrease inflammation in *S. typhimurium* infection in neonatal broiler chicks	*In vivo* (Newly hatched chicks)	-	[172]
miR-135b, miR-155 miR-26b and miR-18a	*Lactobacillus acidophilus* and *Bifidobacterium bifidum (Bla/016P/M)*	1 × 10^9^ CFU/g and 1 × 10^9^ CFU/g	Up regulation of miR-135b, miR-155 and down regulation of miR-26b, miR-18a	APC, PTEN, KRAS, and PU.1	-.	*In vivo* (Mice)	-	[173]
miR-423-5p	*Enterococcus faecium NCIMB 10415*	3.6 × 10^6^ CFU/g	Up regulation of miR-423-5p	Immunoglobulin lambda light C region (IGLC) and immunoglobulin kappa constant (IGKC)	-	*In vivo* (Landrace pigs)	-	[174]
	*Lactobacillus rhamnosus GG, Bifidobacterium animalis subsp. lactis Bb-12* and *L.* *acidophilus La-5*	5 × 10^10^ CFU, 5 × 10^10^ CFU and 5 × 10^9^ CFU				*Human*	54	[175]
miR-122a	*Lactobacillus rhamnosus GG*	1 × 10^9^ CFU	Down-regulation of miR-122a	?	Decrease ethanol-elevated miR122a levels and attenuate ethanol-induced liver injury	*In vivo* (Mice)	-	[176]
miR-146a	*Escherichia coli* Nissle 1917 (EcN) (O6:K5:H1)	NA	Up-regulation of miR-146a in both EPEC and ECN	IRAK1 and TRAF6	Reduce IL-8 as well as CXCL1 in T84 cells	*In vitro* (Human epithelial and THP-1 cells)	-	[177]
miR-21, miR-92a, miR-155, miR-663	*Lactobacillus* *acidophilus* (La) ATCC strain 4356	10^9^ – 10^10^ CFU/ml	Up-regulation of miR-21 and down-regulation of miR-155	MiR-92a targets integrin a5, klf-2 and klf-4/ MiR-155 targets AtR1 and Ets-1/ MiR-663 targets VEGF	Reduce apoptosis, necrosis and inflammatory	*In vitro*(HUVEC)	-	[178]
miR-155, miR-223, miR-150, miR-375 and miR-143	*Lactobacillus fermentum* CECT5716 and *L. salivarius* CECT5713	5 × 10^8^ CFU	Down-regulation of miR-155, miR-223 and miR-150 and up-regulation of miR-143	c-Myb (miR-150)	Reduce expression of pro-inflammatory cytokine IL-1β	*In vivo (Mice)*	-	[179]
miR-203, miR-483-3p and miR-595	*Escherichia coli* Nissle 1917 (EcN)	NA	Up-regulation of miR-483-3p and down-regulation of miR-203 and miR-595	miR-203 targets tight junction protein ZO-2/ miR-595 targets Mucin-4-precursor/ miR-483-3p targets beta-defensin 111 precursor	Improve effects of EcN.	*In vitro* (T84 epithelial cells)	-	[180]
miR-21 and miR-200b	*Leuconostoc mesenteroides*	NA	Down-regulation of miRNA-21 and miRNA-200b	NF-kB inhibitory subunit (IKB) and RelA	Induce apoptosis in colon cancer cell line by up-regulation of MAPK1, Bax, and caspase 3, and down-regulation of AKT, NF-kB, Bcl-XL	*In vitro* (HT-29 cells)	-	[181]
miRNA-29a miRNA-29b miRNA-29c	A mixture of*: Lactobacillus plantarum* DSM 24730*, Streptococcus thermophiles* DSM 24731*, Bifidobacterium breve* DSM 24732*, L. paracasei DSM* 24733*, L. delbrueckii* subsp*. Bulgaricus* DSM 24734*, L. acidophilus* DSM 24735, *B. longum* DSM 24736, and *B. infantis* DSM 24737	1.8 × 10^9^ CFU	Unchanged	-	-.	*Human*	10	[182]
miR-146a and miR-155	*Lactobacillus rhamnosus GG* (LGG) and *L.* *delbrueckii* subsp. *Bulgaricus* (*L.del*)	NA	Up-regulation of miR-155 and down regulation of miR-146a.	-	Down-regulation of p38 while IκB expression was significantly decreased in L. del-treated DCs.	*In vitro* (Human monocyte-derived dendritic cells)	-	[183]
miR-143, miR-150, miR-155, miR-223, and miR-375	*Escherichia coli* Nissle 1917 (EcN)	5 × 10^8^ CFU	miR-150, miR-155, and miR-223 were up-regulated. miR-375 was down-regulated.	Housekeeping gene (SNORD95)	The intestinal anti-inflammatory effects of EcN were associated with altered gut microbiome in mouse experimental colitis.	*In vivo* (Mice)	-	[184]
miR-148a	*Bifidobacterium bifidum MIMBb75 B. bifidum NCC390* or *B. longum NCC2705*	1 × 10^8^ CFU	Up-regulation after 1 and 4 hours, but not after 24 h (in vitro) Up-regulation after 2 but not 14 days (in vivo)	EPAS1	Reduce EPAS1 expression in Caco-2 cells and mouse cecum.	*In vitro* (Caco-2 cells) *and In vivo* (Mice)	-	[185]
miR-744	*Lactobacillus crispatus* 2743 and *L. gasseri* 3396	5 × 10^7^ CFU/ml	Up-regulation	ARHGAP5	Trigger lactic acid-induced migration and invasion in SiHa cells.	*In vitro* (SiHa cells)	-	[186]
Let-7b	Encapsulated mixture of *Lactobacillus plantarum, L. acidophilus* -11 and *Bifidobacterium longum*-88 or encapsulated mixture of *Enterococcus faecium* and *Bacillus subtilis*	2.6×10^14^ CFU in first mixture and 5.0×10^8^ CFU in second mixture	Down-regulation	HMGA2 FIGN MAPK6 ARID3B	-	*Human and In vitro* (NCM460 cells)	79	[187]

In one study by Heydari et al. supplementation with *Lactobacillus acidophilus* and *Bifidobacterium bifidum* probiotics in a mouse model of azoxymethane (AOM)-induced colon cancer was investigated. The results showed that the expression levels of miR-135b, miR-155, and KRAS (one of the target genes of these miRNAs) increased after azoxymethane cancer induction, and administration of a probiotic preparation containing *Lactobacillus acidophilus* and *Bifidobacterium bifidum* decreased the above-mentioned factors. Conversely, cancer induction with azoxymethane reduced the expression of miR-26b, miR-18a, APC, PU.1, and PTEN in mice, and probiotic supplementation increased them again. It seems that Lactobacillus acidophilus and Bifidobacterium bifidum though increasing the expression of the tumor suppressor miRNAs and their target genes and decreasing the oncogenes can improve colon cancer treatment [173].

*Enterococcus faecium NCIMB 10415* is a probiotic species that has been shown to affect the intestinal microbial flora, and improve the immune system response in numerous human and animal studies [188, 189]. In one *in vitro* study using next-generation sequencing, Kreuzer-Redmer and colleagues analyzed the differential expression of the miRNAs and potential target genes in the ileal and jejunal lymphatic tissue isolated from piglets which had been fed with *E. faecium* NCIMB 10415 versus control animals. They found that feeding *E. faecium* affected the expression of miR-423-5p as well as regulating its target gene IGLC. Therefore, *E. faecium* benefits the immune cells in the small intestine probably by affecting the expression of miR-423-5p [174].

*Escherichia coli Nissle 1917* (*EcN*) is a non-pathogenic Gram-negative bacterium of the Enterobacteriaceae family, which when used as a probiotic, has beneficial effects on human health [190]. In one in vitro study, *E. coli* (*EPEC*) pathogenic strain E2348/69 and *E. coli Nissle 1917* (*EcN*) were tested in human T84 and THP-1 cells to compare the effects of the two strains on cytokine and miRNA expression. EcN increased the expression of CXCL1 and IL-8 in human T84 epithelial cells infected from the basolateral side. miR-146a is a molecular adaptor in the Toll-like receptor (TLR)/NF-κB signaling pathway. In this study, miR-146a was increased in T84 and THP-1 cells treated with EPEC, but this increase was less pronounced when these cells were incubated with EcN. Two miR-146a target genes were also identified, including IRAK1 and TRAF6. So, the probiotic *EcN* induced the expression of miR-146a in epithelial and immune cells, though this induction was reduced by incubation with pathogenic strain EcN [177].

miR-122 is the most frequent miRNA found in the liver, and plays an important role in liver biology and the pathogenesis of liver diseasess [191]. MiR-122 can down-regulate the proliferation and transactivation of hepatic stellate cells (HSCs) which play a role in liver fibrosis [192]. It is well known that alcoholic liver disease (ALD) causes a great burden of morbidity and death. In fact, persistent use of alcohol affects the homeostasis of the intestinal microflora, increases endotoxemia, disrupts the intestinal cell tight junction barrier, and causes liver steatosis or steatohepatitis. It was shown that a bacteria-free LGG culture supernatant (LGGs) and probiotic *Lactobacillus rhamnosus* GG (LGG) both promoted intestinal epithelial integrity and protected intestinal barrier function in ALD. Nonetheless, there is insufficient information on the mechanism of action of LGGs for increasing intestinal tight junction proteins. Another study conducted by Zhao et al. found that chronic ethanol exposure could increase the expression of the intestinal miR122a and decrease the expression of occludin, causing increased intestinal permeability. Supplementation with LGGs decreased the levels of miR122a caused by ethanol administration, and lessened ethanol-induced liver damage in mice. Over-expression of miR122a in a Caco-2 cell mono-layer caused a remarkable reduction in the level of occludin protein, similar to ethanol exposure. On the contrary, inhibiting miR122a increased the expression of occludin. It was suggested that a LGG supplementary regimen could affect intestinal integrity *via* inhibiting miR122a, thereby restoring levels occludin in the mice subjected to chronic ethanol intake [176].

Infection with high-risk human papillomavirus (HPV), and subsequent genomic integration leads to cervical cancer. Notably, E6 and E7 oncogenes are expressed leading to immortalizing the cells, and the effects on cell migration and invasion predispose to tumor metastasis [193]. Nonetheless, there is insufficient data on the fundamental mechanisms underlying this process, including the movement of the cervical cancer cells into the tumor micro-environment. In addition, lactic acid has been commonly viewed as an important constituent of the tumor microenvironment, as it is a by-product of glycolysis. In fact, researchers have suggested that the lactic acid content in primary malignant lesions could be a prognostic marker for the patient overall survival and risk of metastasis [194]. Based on some studies, there was a correlation between higher amounts of lactic acid and the risk of metastasis and poor prognosis in head and neck squamous cell carcinoma, rectal adenocarcinoma and cervical cancer [194, 195]. However, little attention has been paid to the biology of lactic acid in cervical cancer. For instance, Li et al. assessed the effects of lactic acid on the invasion and migration of the HPV16 positive SiHa cells. It was found that the addition of extra-cellular lactic acid reduced the expression of E7 and 16 HPV-16 E6 oncogenes, and increased the migration and invasion of SiHa cells by up-regulating miRNA-744 levels. This research added further knowledge about the relationship between lactic acid, cell migration and invasion, and implicated the miR-774 in the mechanism [186].

In a study by Liu et al., a study population of 186 patients was recruited who underwent surgery for colorectal cancer. The intervention group (n=93) received either an encapsulated mixture of *Lactobacillus plantarum* (CGMCC No.1258)*, L. acidophilus-11* and *Bifidobacterium longum-88* or an encapsulated mixture of *Enterococcus faecium* and *Bacillus subtilis,* while the placebo group (n=93) received maltodextrin capsules. The intervention group showed reduced levels of complications and infection, accompanied by increased serum and tissue levels of the miRNA let-7b. In this study, P38 MAPK was identified as a target gene for let-7b using NCM460 cells. Therefore, it could be suggested that mixture of probiotics used in this study reduced the complications and infection after surgery for colorectal cancer probably through increasing the level of the miRNA let-7b [187].

The mir-148/mir-152 family is composed of three highly conserved mature miRNAs with similar sequences, structures and the same core region (i.e., UCAGUGCA). This family includes mir-148a, mir-148b and mir-152. The precursor mir-148/mir-152 has a stem-loop structure which is cleaved by a series of enzymes in the nucleus and cytoplasm to form mir-148a, mir-148b and mir-152 sequences [196]. In one study, the miRNAs in Caco-2 cells and cecal tissue from mice were evaluated after treatment with *Bifidobacterium bifidum MIMBb75, B. bifidum NCC390* or *B. longum NCC2705* probiotics. After 1 and 4 hours miR-148a was increased in response to *B. bifidum MIMBb75* in Caco-2 cells. In the animal study, Taibi et al. explored whether *Bifidobacterium* strains (including Bifidobacterium bifidum MIMBb75, B. bifidum NCC390 or B. longum NCC2705) could change the expression of the miR-148a and its target gene in the intestines. The expression of miR-148a and its respective validated target EPAS1 in Caco-2 cells and mouse cecum in response to *B. bifidum* NCC390, *B. bifidum* MIMBb75, and *B. bifidum* NCC2705 was evaluated. It was concluded that *in vitro* exposure to *B. bifidum* MIMBb75 (but not *B. bifidum* NCC390 or *B. longum* NCC2705) increased the expression of miR-148a after 1-4 hours (p<0.01) but not after 24 hours. Administration of *B. bifidum* MIMBb75 to C57BL/6J mice enhanced the expression of miR-148a in the cecum after 2 but not 14 days (p<0.05). It was found that increased miR-148a expression was followed by reduced EPAS1 expression in the Caco-2 cells and cecal tissue (p<0.05). Finally, it was shown that silencing miR-148a could reverse the *B. bifidum* MIMBb75-dependent down-regulation of EPAS1 [185].

Another study used *E. coli* Nissle 1917 as a probiotic, and investigated the effects of this strain on the expression of inflammatory cytokines and miRNAs in mice with dextran sodium sulfate (DSS)-induced colitis. Probiotic treatment reduced the secretion of inflammatory cytokines and restored epithelial integrity. Probiotic treatment also restored to normal levels the various types of miRNAs involved in the inflammation process (miR-143, miR-150, miR-155, miR-223, and miR-375). They concluded that probiotics could modulate the expression of miRNAs in the colitis affected mice to ameliorate inflammation and restore gut homeostasis [184].

Toll like receptor 4 (TLR4) is an pathogen recognition receptor (PRR) expressed on many immune cells [197]. The TLR4 signaling pathway plays an important role in triggering the innate immune response in many inflammatory disorders [198]. P38 mitogen-activated protein kinases (MAPKs) are activated in response to many factors, such as inflammatory cytokines. There are four members of the p38MAPK family (p38α, p38β, p38γ and p38δ). The p38MAPK signaling pathway plays a major role in the biosynthesis of inflammatory cytokines, and can be used as a target to treat various disorders of the immune system [199]. One in vitro study investigated the effects of probiotics on immune responses using *Lactobacillus rhamnosus GG* (*LGG*) and *L. delbrueckii* subsp. *Bulgaricus* (*L. del*) as probiotics added to human monocyte-derived dendritic cells (DCs). The use of *LGG* reduced the expression of p38 MAP kinase during the intervention. Also, the expression of IκB was significantly reduced in the *L. del*. group. The probiotic LGG could regulate immune system responses through increasing the level of miR-155 as well as reduction the expression of miR-146a which targets NFκB [183].

**Fig 3:**
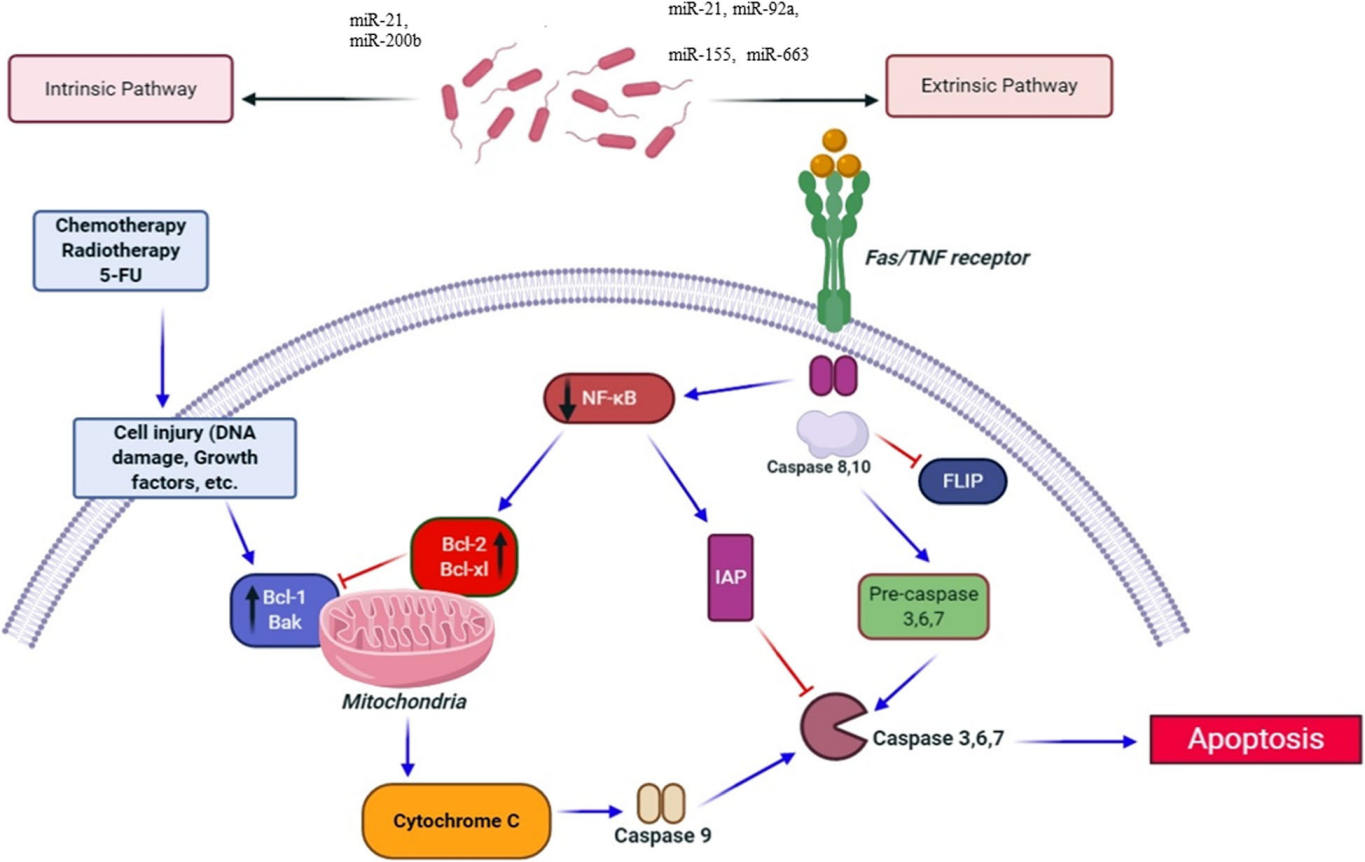
A schema of anti- apoptotic effects of probiotics. Various microRNAs i.e., miR-21, miR-200b and miR-21 can indirectly affect on apoptosis pathways

*Leuconostoc* species are lactic acid bacteria used as fermentation agents in numerous foods, and in the food and beverage industry. *Leuconostoc* spp. naturally produce chemical compounds that have inhibitory properties against other bacteria. The most important of these compounds are called bacteriocins (antibacterial peptides) [200]. One study performed by Vahed et al. investigated the anticancer effects of *Leuconostoc mesenteroides* in colon cancer cells (HT-29). Researchers isolated the *L. mesenteroides* strain from conventional dairy products. HT-29 cells were treated with the conditioned-medium of *L. mesenteroides* bacteria, and apoptosis was examined. This study showed that *L. mesenteroides* medium as a potential adjunctive treatment for cancer could promote apoptosis in the colon cancer cells *via* up-regulating Bax, MAPK1 and caspase 3, and down-regulating NF-κB, AKT, Bcl-XL, probably by reducing the expression of onco-miRNAs, including miR-200b and miR-21 (Fig. 3 )[181].

## Conclusions

The investigation of the interaction between human health and the gut microbiota, is essential for understanding many diseases of the modern world. As the human diet has become steadily more processed, it is thought that the gut is no longer exposed to many bacteria as it has been for most of human evolution. Accumulating data suggests that the administration of probiotics, living microorganisms typical of the healthy human gut, could have a crucial role in the prevention and treatment of many pathological conditions. Probiotics may function through several signaling pathways and by regulating biochemical biomarkers, such as miRNAs. Therefore, evaluation of the possible relationship between probiotics and miRNAs is important to discover the underlying role of gut microbiota in human health. Regarding the concept of the application of probiotics in different foods, such as yoghurt, milk and cheese, and their use as supplements to the human diet, future investigations should focus on the role of miRNAs, and further animal studies and clinical trials will be required before clear guidelines can be laid down.

## Data Availability

The primary data for this study is available from the authors on request.

## Abbreviations

5-ASA: 5-aminosalicylic acid
AD: Atopic dermatitis
AGE: Acute gastroenteritis
Ago2: Argonaute 2
ALD: Alcoholic liver disease
AOM: Azoxymethane
APC: Adenomatous polyposis coli
AR: Adequate relief
AST: Aspartate transaminase
B: *Bifidobacterium*
Bcl-XL: B-cell lymphoma-extra large
BL: *Bifidobacterium longum* NCC3001
BL: *Bifidobacterium longum*
BMI: Body mass index
CD: Celiac disease
CFU: Colony-forming unit
CP: Chronic periodontitis
CRC: Colorectal cancer
CRF: Corticotropin-releasing factor
CXCL1: Chemokine (C-X-C motif) ligand 1
DAO: Diamino-oxidase
DC: Dendritic cells
dsRNA: Double-stranded RNA
DSS: Dextran sodium sulfate
*E. faecium*: Enterococcus faecium
ECN; *EcN*: *Escherichia coli Nissle*
EN: Enteral nutrition
EPAS1: Endothelial PAS domain-containing protein 1
EPEC: *Escherichia coli*
ERAS: Enhanced recovery after surgery
FE: Fiber-enriched
FEP: Fiber- and probiotic-enriched
FF: Fiber-free
GC: Gastric cancer
GFD: Gluten-Free Diet
GIT: Gastrointestinal tract
HAMA: Hamilton Anxiety Scale
HOMA-B: Homeostatomy model assessment-B cell function
HPV: Human papillomaviruses
hs-CRP: High-sensitivity C -reactive protein
HSC: Hepatic stellate cells
IBD: Inflammatory bowel disease
IBS-C: IBS symptoms related to constipation
IBS: Irritable bowel syndrome
IFN-γ: Interferon gamma
IGKC: Immunoglobulin kappa constant
IGLC: Immunoglobulin Lambda Constant 1
IGLC: Immunoglobulin lambda light C region
IL-1β: Interleukin-1beta
IL: Interleukin
KRAS: Kirsten rat sarcoma viral oncogene
L: *Lactobacillus*
*L.del*: *Lactobacillus delbrueckii*
La: *Lactobacillus acidophilus*
*LcS*: *Lactobacillus casei* strain *Shirota*
LGG: *Lactobacillus rhamnosus GG*
LOHS: length of hospital stay
LP: *Lactobacillus paracasei*
LPR: *Lactobacillus rhamnosus*
LPZ01: *Lactobacillus plantarum* Z01
LR: *Lactobacillus rhamnosus*
MAPK: Mitogen-activated protein kinase
miRNAs: MicroRNAs
MTX: Methotrexate
MYB: Myeloblastosis
NEC: Necrotizing enterocolitis
NF-κB: Nuclear factor kappa-light-chain-enhancer of activated B cells
ODC: Ornithine decarboxylase
PCR: Polymerase chain reaction
Pri-miRNAs: Primary miRNAs
PRR: Pathogen recognition receptor
PTEN: Phosphatase and tensin homolog
PUT: Putrescine
RA: Rheumatoid arthritis
RCT: Randomized controlled trial
RISC: RNA-induced silencing complex
RLC: RISC-loading complex
SGA: Subjects' global assessment
SRP: Scaling and root planning
TLR: Toll-like receptor
TNFα: Tumor necrosis factor alpha
Tr1: T-regulatory 1
TRBP: Transactivation response element RNA-binding protein
UC: Ulcerative colon
UTI: Urinary tract infection
XPO5: Exportin 5
